# Hybrid-type endoscopic thyroidectomy (HET: Tori’s method) for differentiated thyroid carcinoma including invasion to the trachea

**DOI:** 10.1007/s00464-013-3245-4

**Published:** 2013-11-22

**Authors:** Masayuki Tori

**Affiliations:** 1 Department of Endocrine Surgery, Osaka Police Hospital, 10-31 Kitayama-cho, Tennouji-ku, Osaka, 543-0035 Japan; 2 Department of Surgery, Osaka Police Hospital, 10-31 Kitayama-cho, Tennouji-ku, Osaka, 543-0035 Japan

**Keywords:** Endoscopic thyroidectomy, Thyroid cancer, Tracheal invasion, Hybrid-type operation, HET (Tori’s method)

## Abstract

**Background:**

Endoscopic thyroidectomy (ET) or robotic thyroidectomy is yet to be applied to thyroid carcinoma invasive to the trachea and to wide lymph node node metastasis. On the other hand, small-incision thyroidectomy lacks sufficient working space and clear vision. The author has newly developed hybrid-type endoscopic thyroidectomy (HET) to overcome these problems.

**Methods:**

From March 2011 to February 2012, HET was performed for 85 patients. Clinicopathologic characteristics were analyzed. To evaluate the superiority of HET for malignancy representatively, conventional lobectomy with central compartment node dissection (CCND) performed 1 year previously was compared with HET. In lobectomy and node dissection, a single skin incision (1.5 cm) is made above the clavicle, with a port incision (5 mm) made 3 cm below the clavicle. Then CCND is performed directly through the incision by lifting up the isthmus. To obtain sufficient working space for the lobectomy, the strap muscles are taped and pulled toward the head, then hung by the cradle. The thyroid lobe is retracted to the midline with a retractor, followed by isolation of the inferior laryngeal nerve and transection of the inferior thyroid vessels with the monitor of the scope. Lateral lymph nodes dissection can be performed at the same time, if necessary. In total thyroidectomy, the same procedure is performed at the opposite side. The scalpel can be used to shave through each incision in case of tracheal invasion.

**Results:**

Of the 85 cases, 62 were malignant, involving papillary thyroid carcinoma (PTC), and 23 were benign. Total thyroidectomy was performed for 22 of the PTC cases and CCND for 49 of the cases. Shaving for tracheal invasion was performed for eight patients. No mortality, complications, recurrence, or metastasis was found 1–2 years after the operation. Compared with conventional thyroidectomy, HET was superior in blood loss, visual analog scale, and postoperative hospital stay.

**Conclusion:**

The author’s method (Tori’s method) might be less invasive, cosmetically excellent, and moreover, safe and feasible for differentiated thyroid carcinoma including invasion to the trachea.

Since the first performance of endoscopic parathyroid surgery by Gagner [1] and report of endoscopic thyroid lobectomy by Hüscher et al. [2], endoscopic thyroidectomy (ET) has been used via multiple approaches including the anterior chest [3, 4], the axilliary fossa [5, 6], the breast [7], the submandibular area [8], and combinations of these [9, 10].

Recently, an experimental trial of the transoral approach was examined [11]. It seems that ET has not been fully accepted, although it has been reviewed as cosmetically excellent, especially for young female patients. The defects of ET are that it usually involves technical difficulties (instrumentation and narrow working space), it might not be minimally invasive surgery because it usually requires a much larger area of damage in subcutaneous space and involves severe pain for the patients, and it cannot be applied to advanced differentiated thyroid carcinoma because sufficient lymph node (LN) dissection or shaving of the trachea is impossible.

Robotic thyroidectomy is an emerging new technique, and some reports appreciate its merits [12, 13]. However, it might not be applied for advanced differentiated thyroid carcinoma without improvement in robotic systems and instrumentation [14–16]. In addition, it is associated with a cost problem [16].

On the other hand, small-incision (3–5 cm) surgery has the problem of an operative window and isolation of the back of the thyroid bed because of an incomplete view. Therefore, I have newly developed hybrid-type ET (HET), which is a reasonable mixture of endoscopic surgery and small-incision surgery. I present this novel method as a safe and less invasive procedure for differentiated thyroid carcinoma including widely spreading LN metastasis and tracheal invasion.

## Patients and methods

This study was approved by the Institutional Review Board (IRB) of Osaka Police Hospital. We established and began HET in March 2011. The eligibility criteria for HET specify thyroid nodules smaller than 40 mm (malignant) and smaller than 50 mm (benign) in diameter, no prior surgery to the neck, and no invasion to the mucosa of the trachea. Graves disease is excluded.

I retrospectively reviewed all thyroid surgical patients within 1 year after we began HET (*n* *=* 115) and identified 85 patients who underwent HET for thyroid cancer from March 2011 to February 2012 (for 1 year). None of the patients indicated for the criteria had undergone conventional thyroidectomy. In HET, 62 patients were found to have malignancy (papillary carcinoma), and the others were found to have benign tumor. The benign tumors consisted of 15 follicular adenomas, seven adenomatous hyperplasias, and one cyst.

Clinical examinations performed for each patient included age, sex, body mass index (BMI), tumor size, type of operation, operative time, intraoperative blood loss, postoperative complications, time of hospitalization, and histologic data involving tumor-node-metastasis (TNM) stage. Postoperative pain evaluated 72 h after the operation by a visual analog scale (VAS) ranging from 0 to 10 was recorded in a database.

Operative procedures for the proven cases of thyroid carcinoma were preoperatively determined according to the Japanese guideline for surgical treatment of thyroid papillary carcinoma [17]. Total thyroidectomy was applied to those patients with significant extrathyroid extension and/or clinical node metastasis and/or distant metastasis detected by preoperative radiologic imaging or histologic examination. Lateral neck LN dissection was performed only for the selected cases in which preoperative imaging suggested LN metastasis.

To examine the superiority of HET representatively, those patients with a diagnosis of papillary thyroid carcinoma (PTC) who had undergone conventional lobectomy with central compartment node dissection (CCND) 1 year previously (from March 2010 to February 2011) were examined at the same time (*n* = 36).

### HET surgical procedure

The surgical team is composed of the surgeon and one assistant. Under general endotracheal anesthesia, the patient is placed in supine position with the arms tucked close to the side. A folded pillow is placed vertically under the patient’s shoulders to extend the head and neck slightly.

**Fig. 1:**
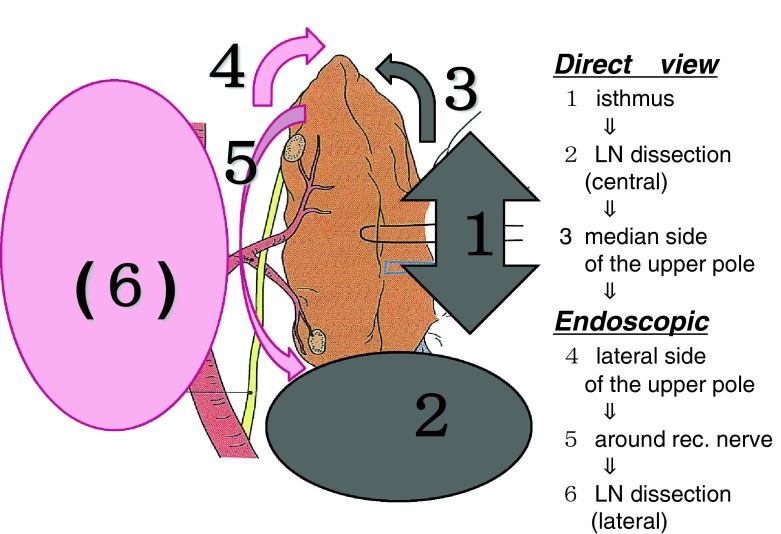
Illustration of the entire procedure. The first half of the procedure treats the middle side of the thyroid. The isthmus is ligated and pulled up toward the head, and then the central compartment node dissection is completed. The latter half of the procedure treats the lateral side of the thyroid with the endoscope. Identification of the recurrent laryngeal nerve and parathyroid can be easily performed

A flowchart of the procedure is shown in Fig. 1. A small-color incision (1.5 cm) is made above the clavicle at the side of the tumor lesion 1.5 cm from the midline when lobectomy and CCND are performed. The same incision is made at the opposite side when total thyroidectomy is performed. The surgical instruments include a 5-mm 30° fiber-optic scope, one 5-mm trocar (EZtrocar, Hakko, Japan), and LigaSure V20 (Covidien, Mansfield, MA, USA).

The platysma is incised, and the wide subplatysmal space is dissected bluntly. The cervical linea alba is divided longitudinally as far up as the thyroid cartilage. Then the anterior border of the sternocleidomastoid muscle is separated from the sternohyoid muscle and the omohyoid muscle. The isthmus of the thyroid is dissected from the trachea ligated on the midline, and by lifting of the thread for ligation, central LN dissection can be performed safely under direct vision, and sometimes with an endoscope.

**Fig. 2:**
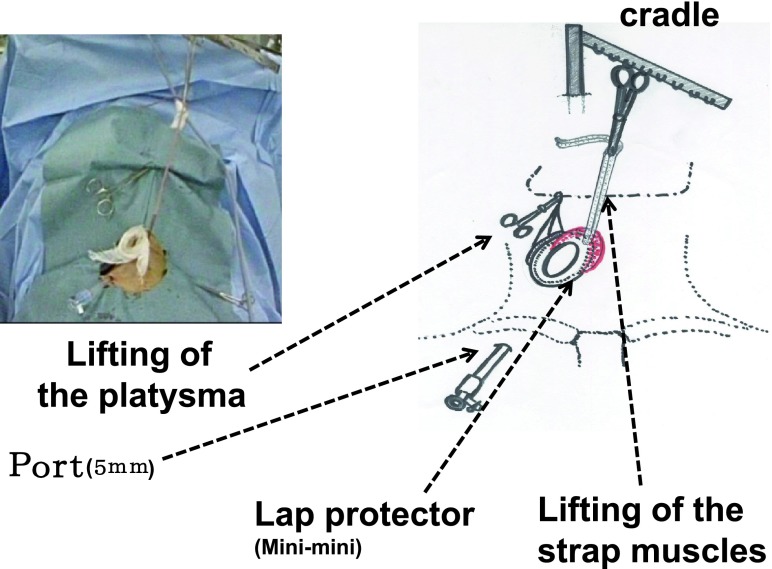
Setting of the endoscopic procedures for the operation (picture and schema). Taping of the strap muscle is hanged on the cradle. A very small protector is inserted through the small-color incision

**Fig. 3:**
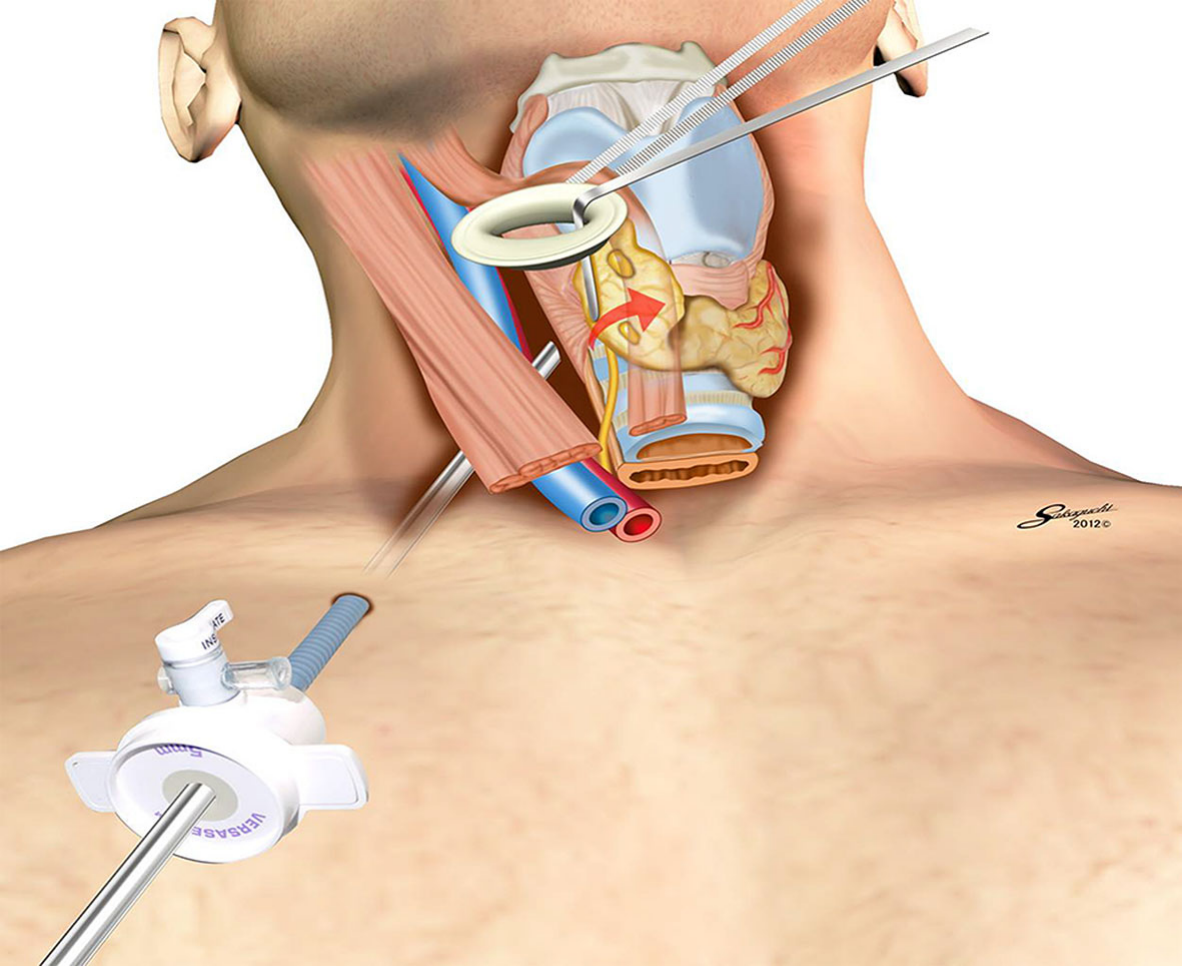
Making room for working space and lighting the recurrent nerve. The muscle flap creates a tent-like working space and provides adequate room for treating the lateral and back sides of the thyroid by retraction of the thyroid using a surgical L-shape retractor. The recurrent nerve is clearly identified by making room for working space and lighting the space

Next, the isthmus is divided at the middle. After the medial side of the thyroid’s upper pole is exposed, the thyroid gland is dissected and detached laterally from the thyroid cartilage, with the external branch of the superior laryngeal nerve identified and preserved. The smallest lap protector (1.5–2 cm; minimini, Hakko, Japan) is attached to the incision, after which the overlying strap muscles are taped and lifted up by the clamp fixed to the cradle (Fig. 2). The muscle flap creates a tent-like working space and provides adequate room to treat the lateral and back sides of the thyroid by retracting the thyroid using the surgical L-shaped retractor (Fig. 3).

A 5-mm endoscopy port is placed 3 cm below the clavicle and at the lateral side of the 1.5-cm incision, which gets through the sternocleidomastoid muscle to spotlight the lateral side of the thyroid lobe and clearly magnify structures such as the recurrent laryngeal nerve and parathyroid glands. Under endoscopic vision, the affected thyroid lobe is pulled medially using the retractor, and the sheet between the thyroid and the carotid artery is opened to reach the vertebral plane. Consequently, the recurrent nerve and parathyroid glands can be identified easily. The vessels of the superior pole are sectioned using LigaSure V20.

The middle thyroid vein also is sectioned by LigaSure V20, and the inferior thyroid artery is exposed. The recurrent laryngeal nerve and a parathyroid gland are identified. The branches of the inferior thyroid artery are sealed and sectioned on the thyroid capsule. The thyroid is freed from the trachea by gentle pulling medially with the retractor. Instruments such as conventional electric scissors can be used during the procedure.

**Fig. 4:**
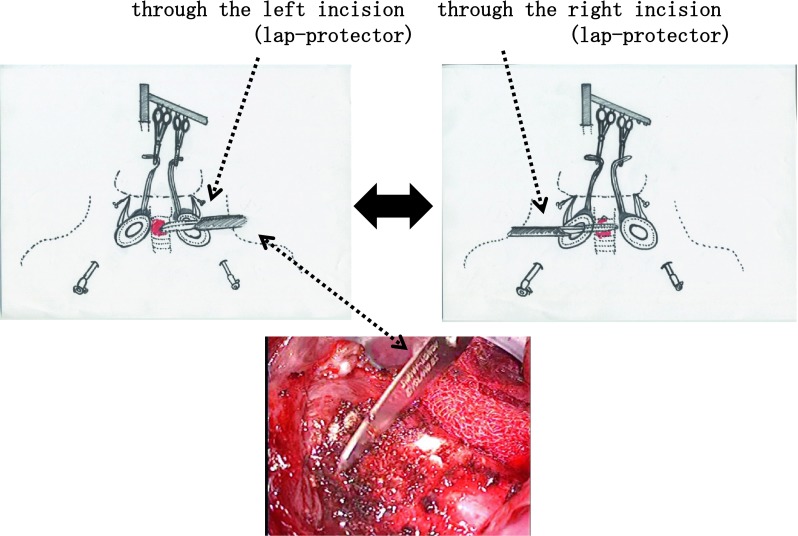
Shaving of the trachea. When tracheal invasion is encountered, total thyroidectomy should be performed, leaving the invasive lesion. The scalpel then can be used through each port to remove the lesion

The same procedure is followed to remove the opposite lobe after a similar incision is made symmetrically at the opposite side when total thyroidectomy is performed. If lateral compartment LN dissection is necessary, it can be performed easily by retracting the carotid sheath medially. Even if the tumor invades the trachea, after near total thyroidectomy is performed beside the invasion area, shaving can be performed through either incision (lap protector) using the scalpel (Fig. 4).

A blake drain is inserted for drainage through the 5-mm trocar hole. Autotransplantation of one or two parathyroid glands usually is performed. The parathyroid tissue is cut into 1–2-mm pieces and placed in small pockets within the sternocleidomastoid muscle. The 1.5-cm wound is closed with absorbable sutures as in open thyroidectomy. All the operations, including the endoscopic and conventional procedures in the current series, were performed by a single surgeon.

### Assessment of postoperative pain

Postoperative pain was assessed by means of VAS 72 h after the operation (on day 3). The VAS is a tool widely used to measure pain. A patient is asked to indicate his or her perceived pain intensity, most commonly along a 10-cm horizontal line, and this rating then is measured from the left edge.

### Statistical analysis

Continuous variables were compared using the Mann–Whitney *U* test. Categorical variables were compared using Yates’ *χ* ^2^ test or Fisher’s exact test as appropriate. Continuous variables are reported as mean ± SD. Categorical variables are reported as number and percentage. Statistical analysis was performed using Excel 2007 (Microsoft, Redmond, Washington, USA) with the add-in software yStat 2008 (Shinya Yamazaki, Koriyama, Japan). All tests of significance were two-sided, and *p* values lower than 0.05 were considered significant.

## Results

Between March 2011 and February 2012, 85 patients (66 women and 19 men) underwent ET with HET. The data are expressed as mean ± SD. The age of the patients was 56.2 ± 14.2 years (range 28–83 years), and the tumor size was 20.5 ± 13.1 mm (range 4–60 mm). The postoperative pathology reported the diagnosis as papillary thyroid carcinoma in 62 cases and as benign tumor in 23 cases. The carcinomas were stage 1 in 26 cases, stage 2 in one case, stage 3 in 28 cases, and stage 4a in seven cases. The postoperative hospital stay was 5.07 ± 1.14 days (range 3–7 days).

No conversions from endoscopic to open thyroidectomy were performed. No complications occurred in the postoperative course, and there was no postoperative mortality (Table 1).

**Table 1:** Demographic findings for the 85 patients who underwent hybrid-type endoscopic thyroidectomy (HET)

Clinicopathologic characteristic	Value
Age, years (range)	56.2 ± 14.2 (28–83)
Sex (male/female)	19/66
BMI, kg/m^2^ (range)	1.575 ± 0.176 (1.264–2.144)
Tumor size, mm (range)	20.5 ± 13.1 (4–60)
Operation time, min (range)	135.3 ± 54.5 (48–350)
Blood loss, ml (range)	1.06 ± 5.74 (0–50)
Lymph node retrieval count, *n* (range)	
Central compartment (*n* = 62)	6.86 ± 3.96 (1–20)
Lateral compartment (*n* = 13)	8.62 ± 7.26 (2–23)
Pathologic classification	
Malignancy	
Papillary thyroid carcinoma	62
Benign	
Follicular adenoma	15
Adenomatous hyperplasia	7
Cyst	1
TNM stage (for papillary thyroid carcinoma)	
Stage 1	26
Stage 2	1
Stage 3	28
Stage 4a	7
Postoperative hospital stay: days (range)	5.07 ± 1.14 (3–7)
Complications^a^ and mortality	0

*BMI* body mass index, *TNM* tumor-node-metastasis

^a^Complications exclude temporary recurrent nerve palsy

The types of operation performed were lobectomy alone (*n* = 16), lobectomy with CCND (*n* = 35), lobectomy with modified radical neck dissection (MRND) (*n* = 5), total thyroidectomy alone (*n* = 2), total thyroidectomy with CCND (*n* = 9), total thyroidectomy with CCND and shaving of the trachea invaded by the tumor (*n* = 5), total thyroidectomy with MRND (*n* = 5), total thyroidectomy with MRND and shaving of the trachea invaded by the tumor (*n* = 3), and partial resection (*n* = 5). Among the total thyroidectomies with MRND, partial esophagectomy with direct suture was performed in two cases.

The median operation times were 110 min (range 55–150 min) for lobectomy alone, 130 min (range 68–213 min) for lobectomy with CCND, 134 min (range 108–199) for lobectomy with MRND, 65.74 min for total thyroidectomy alone, 186 min (range 106–282 min) for total thyroidectomy with CCND, 170 min (range 134–350 min) for total thyroidectomy with CCND and shaving of the trachea invaded by the tumor, 207 min (range 139–285) for total thyroidectomy with MRND, 144, 145, and 168 min for total thyroidectomy with MRND and shaving of the trachea invaded by the tumor, and 78 min (range 69–99) for partial resection. The operation time included setting up of the endoscopic instruments and parathyroid autotransplantation, which is routinely performed for malignant cases even in the lobectomy (Table 2). In terms of postoperative complications, no permanent hypocalcemia occurred. Three cases of transient recurrent nerve palsy (3.90 %) were found in the conventional group, but no permanent palsy was found in either group (Table 3).

**Table 2:** Classification and operation time for the hybrid-type endoscopic thyroidectomy (HET) procedures

Type of operation	*n*	Time Min (range)
Lobectomy alone (for benign tumor)	16	110 (55–150)
Lobectomywith CCND	35	130 (68–213)
Lobectomy with MRND	5	134 (108–199)
Total thyroidectomy alone (for benign tumor)	2	65, 74
Total thyroidectomy with CCND	9	186 (106–282)
Total thyroidectomy with CCND and shaving	5	170 (134–350)
Total thyroidectomy with MRND	5	207 (139–285)
Total thyroidectomy with MRND and shaving	3	144, 145, 168
Partial resection(for benign tumor)	5	78 (69–99)

*CCND* central compartment node dissection; *MRND* modified radical neck dissection

**Table 3:** Number and rate of postoperative complications in hybrid-type endoscopic thyroidectomy (HET)

Complication	*n* (%)
Permanent hypocalcemia^a^	0/85 (0)
Transient RLN palsy	3/77 (3.90)
Permanent RLN palsy^b^	0/77 (0)
Others	0/85 (0)

*RLN* recurrent laryngeal nerve

^a^Permanent hypocalcemia means hypocalcemia during 3 months

^b^Permanent RLN palsy means palsy during 6 months and excludes the cases with invasion to the trachea and/or recurrent nerve

For a precise evaluation of HET’s superiority over conventional thyroidectomy, HET with CCND was compared with conventional lobectomy incorporating CCND. The background data such as age, sex, and BMI showed no difference. The operation time was not short in either group. We usually perform autotransplantation of the parathyroid gland. Moreover, manipulation in the procedure is very prudent to avoid postoperative complications.

**Fig. 5:**
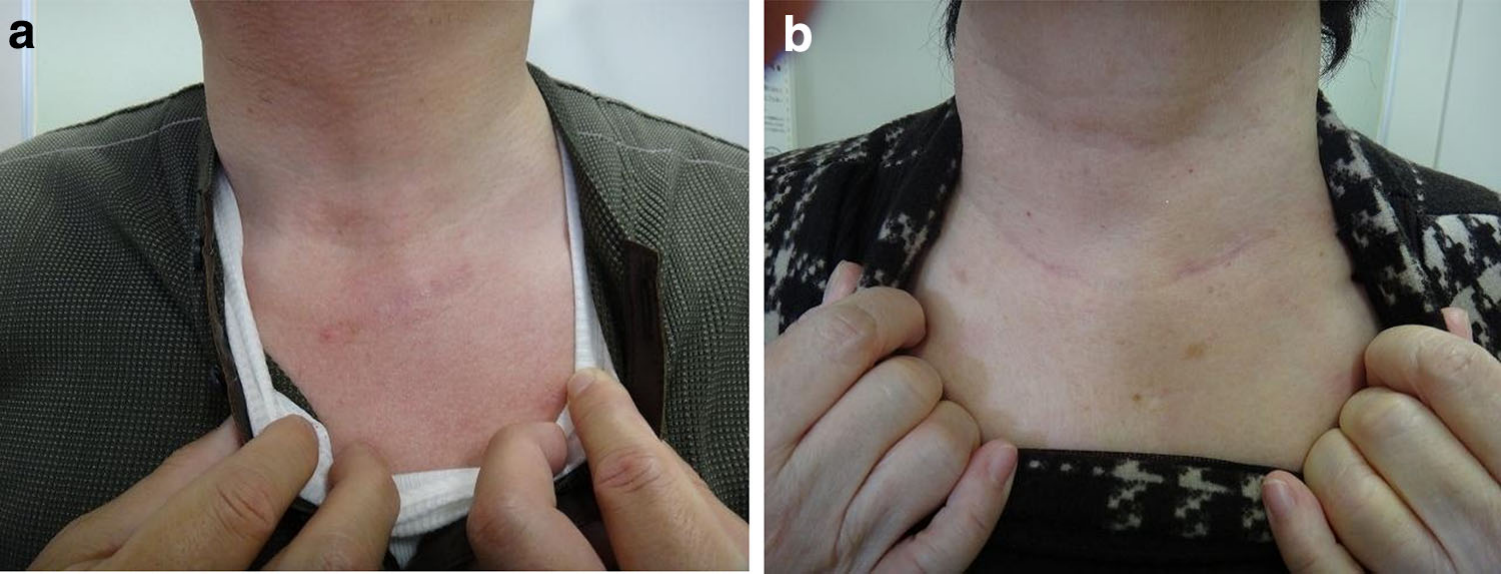
The neck wound 6 months after the surgery. **A** Lobectomy with modified radical neck dissection (MRND). **B** Total thyroidectomy with central component node dissection (CCND)

The operation time did not differ between the two groups. In all the endoscopic cases, the blood loss was 0–10 (very little and almost uncountable), whereas in the conventional group, blood loss was countable. The VAS score was significantly lower in endoscopic group on day 3. Actually, the patients felt no pain at discharge. The postoperative hospital stay was significantly shorter in the endoscopic group. No conversions from endoscopic to open thyroidectomy were performed, and no complications occurred in the postoperative course (Table 4). All the patients treated with this method were very satisfied with the cosmetic results. No extra treatment for the operative scar was necessary in plastic surgery (Fig. 5). No recurrence or metastasis was found in any case 1–2 years after the operation.

**Table 4:** Comparison of hybrid-type endoscopic thyroidectomy (HET) and conventional lobectomy with central compartment node dissection (CCND)

Type of operation	Endoscopic (*n* = 35)	Conventional (*n* = 36)	*p* value
Age, years (range)	59 (28–83)	62 (28–88)	0.121
Sex (male/female)	9/26	6/30	0.520
BMI, kg/m^2^ (range)	1.553 (1.277–1.913)	1.477 (1.161–1.931)	0.073
Tumor size, mm (range)	12 (4–40)	12 (3–40)	0.52
Stage			0.685
1	15	14	
2	0	2	
3	18	16	
4a	2	4	
Operation time, min (range)	130 (68–213)	123 (84–162)	0.161
Blood loss, ml (range)	5 (0–10)	10 (0–100)	0.0078^a^
Central compartment lymph nodes retrieved, *n* (range)	6 (1–20)	6 (1–24)	0.380
VAS scale on day 3, *n* (range)	0 (0–2)	2 (0–4)	0.00016^a^
Postoperative hospital stay, days (range)	5 (3–7)	7 (5–20)	0.00011^a^
Postoperative complications			
Transient RLN palsy	0	4	0^a^
Permanent RLN palsy	0	0	NS
Others	0	0	NS

*BMI* body mass index, *VAS* visual analog scale, *RLN* recurrent laryngeal nerve, *NS* not significant

^a^Significant

## Discussion

Although various endoscopic techniques have been tried worldwide because of their cosmetic benefits, to date, ET has not been standardized for thyroid carcinoma, nor has robotic thyroidectomy, which is becoming popular. Serious technical problems are associated with robotic thyroidectomy. Robotic systems lack tactile sensation and tensile feedback to the surgeon. Prompt open conversion is difficult because the robotic system requires much time for withdrawal of the big arms, making it seriously dangerous to cope with abrupt massive bleeding. The available instruments are only those used for endoscopic surgery and do not include conventional instruments designed for open surgery [18]. Therefore, all the procedures, including robotic surgery are chiefly for benign tumors and usually cannot be applied for malignant cases invasive to adjacent structures. Invasion of the tumor to an adjacent structure such as the trachea or the esophagus is not rare, and more often, invasion to the recurrent laryngeal nerve is encountered during the operation.

The incidence of thyroid cancer itself is increasing, and young females sometimes experience this malignancy. We must therefore take into consideration both the cosmetics and treatment of invasive cases.

With our method, the exclusion criteria for malignancy specify only tumor larger than 4 cm, undifferentiated cancer, and invasion to the mucosa of the trachea. In other words, most cases can be managed by our method, including invasion to the trachea.

Scarless (in the neck) ET (SET) is cosmetically most excellent [2, 19], but theoretically, sufficient LN dissection of the deep mediastinum in the back of the sternum is impossible. By ligating and lifting up the isthmus under direct vision, the methods for central compartment node dissection are similar to those in conventional open thyroidectomy. Moreover, SET itself is not reported to be a minimally invasive technique but a maximally invasive one that involves a longer operative time and greater postoperative pain [20].

Video-assisted neck surgery (VANS) is an excellent method for managing benign tumors [21], although it requires a relatively large incision and working space. LN dissection might be rather difficult, and it is reportedly difficult to dissect the thyroid lobe near the Berry ligament using the harmonic scalpel without causing serious bleeding or recurrent nerve palsy [8]. In ET, especially for procedures around the Berry ligament, laparoscopic instruments are not available because the instruments are mainly designed for abdominal surgery. In fact, when the thyroid is detached from the trachea at the Berry ligament, conventional electric scissors or a scalpel is much better. On the other hand, for dissection of the upper thyroid pole, the vessel-sealing system (LigaSure V20) is excellent because it is short and easy to manipulate. Moreover, we have never experienced rebleeding of the superior thyroid artery.

For all these reasons, a small incision above the clavicle should be necessary, and for manipulating the surroundings of the trachea or esophagus, a “window” for it should be necessary. Therefore, to standardize endoscopic thyroid surgery for malignancy, we abandoned “scarless” procedure in the neck. Both in robotic surgery and with the extracervical (scarless) endoscopic approach, conventional instruments such as scalpels necessary for shaving would never be used, which means that robotic surgery and endoscopic surgery could not be standard methods for advanced thyroid cancers.

Minimally invasive video-assisted thyroidectomy (MIVAT) is an improved method that considers the cosmetics and merits of the small incision [22–26], but the scar in the central lesion of the neck is more conspicuous than the scar in the supraclavicular pouch in our method. Besides the cosmetic problem, MIVAT for thyroid malignancy is controversial [27]. Magnification and clarity of the operation field is needed at the lateral side of the thyroid lobe because of important organs such as the recurrent laryngeal nerve and the parathyroid, but the scope does not reflect these through midline incision. Lateral LN dissection also might be difficult. In contrast to our new method, Miccoli et al. [28] insisted that the inclusion criteria for MIVAT should be strict [26] and that for locally invasive tumors in the presence of LN metastasis, MIVAT must be immediately converted to conventional technique.

Another important thing in ET is finding a way to establish sufficient room for manipulation during the operation. Strap muscles usually are an obstacle, and a way to retract them is a key to making space [29]. We devised HET to make space for manipulation by taping and pulling up the strap muscles. This provides sufficient room for the lateral side of the thyroid lobe and makes it easier to find the recurrent laryngeal nerve, the parathyroid, or both in addition to the merit of lighting [30]. Because the magnification and brightening of HET allows constant sharing of precise anatomic information concerning the recurrent nerve by both the operator and the assistant, the nerve can be treated more carefully. Consequently, temporary recurrent nerve palsy has never been encountered, in contrast to the conventional method.

Another new approach is the method of shaving through the two incisions (lap protector) for tracheal invasion. By using two incisions effectively, the invasive parts can be removed with a conventional scalpel. If a resected specimen cannot be retrieved via the small incision, it can be cut into pieces in the vinyl bag and removed with the bag, or as an option, the port incision can be incised and made longer.

We have overcome the chief defects of conventional thyroidectomy and ET by developing HET, and the perioperative course has been uneventful. However, long-term follow-up evaluation should be done, and further examination for oncologic safety should be performed.

In conclusion, HET with taping and lifting of the strap muscles is an effective and safe method for managing all thyroid differentiated cancers, including invasion to the trachea. The HET procedure has overcome the problems of endoscopic and robotic surgery with minimum scarring. It therefore is safe, cosmetically excellent, and feasible for application to most thyroid cancers.
